# Supplementary material for: Instability of U3Si2 in pressurized water media at elevated temperatures
Source: Commun Chem. 2021 May 13;4:65. doi: 10.1038/s42004-021-00504-3 (PMC9814221; doi:10.1038/s42004-021-00504-3)
Supplement: Supplementary file 1 — Description of Additional Supplementary Files [file 42004_2021_504_MOESM1_ESM.pdf]

Description of Additional Supplementary Files

File Name: Supplementary Data 1

Description: The file contains experimental data collected in the solubility experiments (marked as Experimental data) and the values obtained through thermodynamic calculations for experimental temperatures (marked as Calculated values). Experimental data include TC (experimental temperature, ºC), pH(25) (pH measured after quenching), NaCl, m (content of NaCl in experimental solutions, mol/kg), and U, m (concentrations of U measured in quenched solutions, mol/kg). Calculated values include pH(T) (pH calculated for experimental temperatures), m(HCl) (concentration of HCl in experimental solutions, mol/kg), log fO2 (logarithm of the fugacity of oxygen calculated for experimental conditions, bar), log solubility of UO2 (logarithm of theoretical solubility of UO2, mol/kg), and log solubility of USiO4 (logarithm of theoretical solubility of USiO4).
